# Comparative Efficacy of Four Stain Removal Methods for Bleach-Shade Composite Resins after Immersion in Staining Solutions: An *In Vitro* Study

**DOI:** 10.1155/2023/8909288

**Published:** 2023-06-11

**Authors:** Sedighe Sadat Hashemikamangar, Shakiba Farahani, Shaghayegh Khoshgoo, Parisa Doroudgar

**Affiliations:** ^1^Restorative Departments, Dental School, Tehran University of Medical Sciences, Tehran, Iran; ^2^Dental School, Tehran University of Medical Sciences, Tehran, Iran

## Abstract

**Introduction:**

Bleach-shade composite resins were recently introduced to the market due to the high demand of patients for whiter teeth. This study sought to compare four stain removal methods for bleach-shade composite resins.

**Materials and Methods:**

Seventy-two discs were fabricated from each of the Filtek Z350 XT and Gradia XBW composite resins and immersed in coffee or sour cherry juice staining solutions. Each group was then divided into four subgroups to assess the efficacy of four stain removal methods (finishing with soft-lex disk “brush with pumice” bleaching with carbamide peroxide 16%, bleaching with hydrogen peroxide 40%). The color of each specimen was measured by Easyshade spectrophotometer, and data were analyzed using SPSS 25 statistical package for social sciences.

**Results:**

The home-bleaching technique was more effective than the office-bleaching and pumice for the removal of sour cherry juice stain (*ΔE* = 1.93) and coffee stain (*ΔE* = 3.32) from Gradia composite discs, almost returning the baseline original color. The Sof-Lex discs were more effective than the pumice for the removal of sour cherry juice stain (*ΔE* = 4.11) and coffee stain (*ΔE* = 4.93) from Z350 composite discs but not return the baseline original color.

**Conclusions:**

Filtek Z350 had more discoloration than Gradia Direct. The different materials and solutions reacted differently to the four stain removal methods. In GCJ group after all stain removal methods, *ΔE* reduced to a clinically acceptable level.

## 1. Introduction

Increasing demand for cosmetic dental restorations has led to the development of new resin materials [1]. Most patients prefer shinier and whiter teeth to their own natural teeth [2]. This demand has led to advancements in the bleaching techniques and materials. As a result, bleach-shade composite resins are becoming more popular. These are suitable for use in teeth that have undergone bleaching. The color stability of bleach-shade composite resins plays a fundamental role in their success [3].

Color change of restorative materials after long-term exposure to the oral environment is an important clinical problem. The degree of color change depends on several factors. In the oral environment, esthetic restorations are influenced by the oral hygiene status, consumption of caffeine and colored drinks, tobacco use, and cigarette smoking. In addition to these external factors that cause external discoloration, internal factors such as hydrolysis of the organic matrix or loosening of the filler particles can seriously damage the restorations [4]. Staining of composite resins has been reported following exposure to staining solutions such as coffee, tea, and some other drinks [5]. Discoloration caused by tea occurs as the result of the adsorption of polar coloring agents by the surface of materials, which can be eliminated by toothbrushing, while discoloration caused by coffee occurs due to both adsorption and absorption of coloring agents. Absorption and penetration of coloring agents into the organic phase of restorative materials are probably due to the compatibility of their polymer phase with yellow–brown stains [6]. Among the various cosmetic restorative methods, composite veneers are used more [7]. However, the color stability of composite veneers is a highly debated topic.

Following staining, brushing with a toothpaste and polishing procedures can eliminate all or part of the stains [8]. The polishing procedures can eliminate severe staining of the composite resin. However, they also remove some material from the composite surface [9]. The bleaching toothpastes are attractive and popular, but their abrasive particles can have adverse effects on composite resins. Apart from these, bleaching agents do not seem to be harmful [10]. Vital bleaching techniques are extensively used due to their conservative nature. The modern bleaching agents include hydrogen peroxide and carbamide peroxide as their active ingredients [11].

This study aimed to assess the efficacy of different methods of stain removal from the surface of the bleach-shade composite resins. In this study, the stainability of a bleach-shade microhybrid composite resin and a bleach-shade nanohybrid composite resin was evaluated in sour cherry juice and coffee. Then, the efficacy of four stain removal methods, including polishing with Sof-Lex discs, polishing with pumice paste and brush, bleaching with 16% carbamide peroxide, and bleaching with 40% hydrogen peroxide, was assessed by using a spectrophotometer.

## 2. Materials and Methods

This *in vitro* study evaluated Filtek Z350 XT (3M ESPE, St. Paul, MN, USA) with XWB (extra white body) shade and Gradia Direct Anterior (GC, Corp, Tokyo, Japan) with XBW (extra bleach white) shade composite resins. The minimum sample size was calculated to be 9 in each of the four groups according to a previous study [12] assuming *a* = 0.05, *b* = 0.2, standard deviation of 3.38, and effect size of 0.59, using a one-way analysis of variance (ANOVA) power analysis of PASS 11 software. Cylindrical plexiglass molds with 10 mm diameter and 1 mm height were used for the fabrication of specimens. Composite was applied into the mold, and the mold was compressed between two glass slides with hand pressure in order for the excess material to leak out. The specimens were then cured for 40 s from the superior and inferior surfaces by a curing unit (DTW Lux V; Woodpecker, China) with a light intensity of 1,000–1,100 mW/cm^2^. The intensity of the curing unit was checked using the radiometer at the beginning, and after 24 and 48 specimens were fabricated. The upper surface of specimens was then polished with 1,500, 2,000, and 2,500-grit abrasive papers. The specimens were then immersed in neutral distilled water at room temperature for 24 hr.

The specimens in each group were then randomly assigned to two subgroups for immersion in two staining solutions:

  GCJ: Gradia Direct specimens immersed in sour cherry juice.
  GCO: Gradia Direct specimens immersed in coffee.
  ZCJ: Filtek Z350 specimens immersed in sour cherry juice.
  ZCO: Filtek Z350 specimens immersed in coffee.

### 2.1. Preparation of Staining Solutions

To prepare the coffee solution, 5 g of coffee powder (Nescafe Classic, Nestle, Switzerland) was added to 250 mL of boiling water and then allowed to cool to room temperature. Sour cherry juice was also purchased (San Ich, Iran).

### 2.2. Staining Process

The specimens were immersed in the staining solutions. The specimens were immersed in the solutions at room temperature for 3 hr daily for a total of 24 days. They were suspended so that all sides had exposure to the solution and immersed in neutral distilled water at room temperature between immersion times. The staining solutions were refreshed daily. After completion of the staining period, the discs were rinsed under running water for 1 min. Each group was then assigned to four subgroups for stain removal (*n* = 9).

### 2.3. Stain Removal Process

Group 1: PC: Polishing with pumice powder (Cina, Iran) and brush with a low-speed handpiece for 60 s.

Group 2: SX: Polishing with medium, fine, and superfine aluminum-oxide abrasive discs (SofLex, 3M-ESPE Dental Products, St. Paul, MN, USA) and low-speed handpiece for 20 s by each disc and rinsing after using each disc. Discs are discarded after each use.

Group 3: HB: Daily bleaching with HOME WHITENING (Whitesmile GmbH, Germany) comprising of 16% carbamide peroxide for 12 days, each time for 3 hr. The bleaching gel was applied with a syringe in a thin uniform layer and remained for 3 hr; then the materials were removed from the surface, and the specimens were rinsed with distilled water to remove the bleaching materials completely and dried with a paper towel.

Group 4: OB: Bleaching with POWER WHITENING YF (Whitesmile GmbH, Germany), comprising 40% hydrogen peroxide three times, each time for 20 min in one session. The bleaching was gel applied with an auto-mixed syringe in a thin uniform layer and remained for 20 min; then the materials were removed from the surface, and new gel was applied; after three applications, the specimens were rinsed with distilled water to remove bleaching materials completely and dried with a paper towel.

After each bleaching cycle, the specimens were immersed in neutral deionized water at room temperature.

### 2.4. Color Testing

The color of each specimen was measured at baseline (prior to staining, *T*_0_), after staining (*T*_1_) and after stain removal procedures (*T*_2_). The color of composite discs was analyzed using Vita Easyshade spectrophotometer (VITA Zahnfabrik H. Rauter GmbH & Co., Germany). The color of each disc was analyzed three times and the average of these data was reported as the final result. The color parameters were reported using CIELAB color space relative to CIE standard illuminant D65. All measurements were made with the probe tip of Easyshade spectrophotometer perpendicular to the surface. In order to eliminate the confounding effect of background color on the measurements, a white paper was placed behind the discs. A positioning jig was constructed with an alignment mark so that the center of the device probe was positioned in the center of the specimens each time. The device was calibrated after every five measurements.

The total color change between *T*_0_ and *T*_1_ (*ΔE*_1_) and *T*_0_ and *T*_2_ (*ΔE*_2_) was calculated using the following formula:

(1) $$\begin{array}{c} \Delta E={\left[{\left(\Delta L^{*}\right)}^{2}+{\left(\Delta a^{*}\right)}^{2}+{\left(\Delta b^{*}\right)}^{2}\right]}^{1/2}, \end{array}$$

where *L*^*∗*^ is lightness (−*L*^*∗*^ = black; +*L*^*∗*^ = white), *a*^*∗*^ is green–red (−*a*^*∗*^ = green; +*a*^*∗*^ = red), and *b*^*∗*^ is blue–yellow (−*b*^*∗*^ = blue; +*b*^*∗*^ = yellow).

The data were analyzed using SPSS 25. Univariate ANOVA was applied to assess the effect of type of composite, type of staining solution, and method of stain removal, as well as their interaction effects on the color parameters. In case of significant effect, one-way ANOVA and *t*-test were applied. In case of significant effect determined by one-way ANOVA, pairwise comparisons were performed by Tukey's test. *P* < 0.05 was considered statistically significant. The clinically acceptable color change was defined at *ΔE* = 3.3 [12].

## 3. Results

**Figure 1:**
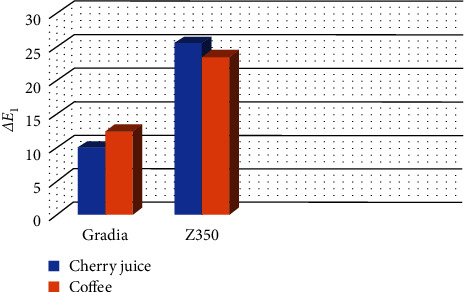
Bar chart of mean color change of two composite resins after immersion in two staining solutions.

Color changes of the two composite resins after immersion in staining solutions compared with baseline (*ΔE*_1_) are presented in Figure 1 and Table 1.

Two-way ANOVA showed that the effect of type of composite on *ΔE* was significant between baseline and after immersion (*P* < 0.001). However, the effect of type of staining solution (*P* = 0.599) was not significant. The interaction effect of type of composite and type of staining solution on *ΔE* was also significant between baseline and after immersion (*P* = 0.046). As shown in Table 1, the color change of Filtek Z350 was greater than that of Gradia Direct.

The color change of Filtek Z350 caused by sour cherry juice was greater than that caused by coffee (ZCJ > ZCO, *P* < 0.001). The color change of Gradia Direct caused by coffee was greater than that caused by sour cherry juice but not significantly (GCJ = GCO, *P* = 0.57).

**Figure 2:**
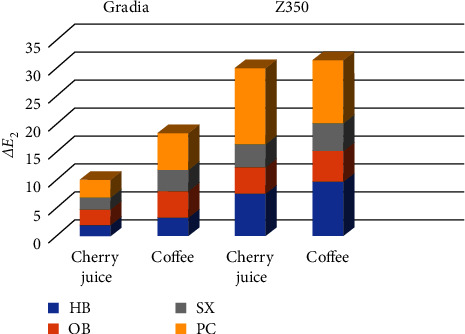
Bar chart of mean color change after four stain removal procedures compared with baseline.

Color change of Gradia Direct after stain removal compared with baseline (*ΔE*_2_) are presented in Figure 2 and Table 2.

According to ANOVA, a significant difference was noted between different stain removal techniques for GCJ between baseline and after stain removal (*P* < 0.001). Tukey's honestly significant difference (HSD) test showed significant differences between HB and OB (*P* = 0.009), and also HB and PC (*P* < 0.001). However, HB and SX were not significantly different (*P* = 0.923). OB and SX were significantly different (*P* = 0.041), while OB and PC had no significant difference (*P* = 0.557). The SX and PC were also significantly different (*P* = 0.001).

According to ANOVA, a significant difference was noted between the efficacy of different stain removal techniques for GCO between baseline and after stain removal (*P* < 0.001). Tukey's HSD test revealed significant differences between HB and OB (*P* < 0.001) and also HB and PC (*P* < 0.001). However, HB and SX were not significantly different (*P* = 0.593). OB and SX were not significantly different either (*P* = 0.151). However, OB and PC (*P* = 0.001) and SX and PC (*P* < 0.001) were significantly different.

Color changes of Filtek Z350 after stain removal compared with baseline (*ΔE*_2_) are presented in Figure 2 and Table 3.

According to ANOVA, a significant difference existed in the efficacy of different stain removal techniques for ZCJ between baseline and after stain removal (*P* < 0.001). Tukey's HSD test revealed a significant difference between HB and PC (*P* < 0.001). However, HB had no significant difference with OB (*P* = 0.136) and SX (*P* = 0.051). OB had a significant difference with PC (*P* < 0.001) but had no significant difference with SX (*P* = 0.965). The SX and PC were also significantly different (*P* < 0.001).

ANOVA showed a significant difference in the efficacy of different stain removal techniques for ZCO between baseline and after stain removal (*P* < 0.001). According to Tukey's HSD test, significant differences existed between HB and OB (*P* = 0.001) and HB and SX (*P* < 0.001). But HB and PC were not significantly different (*P* = 0.389). OB had a significant difference with PC (*P* < 0.001) but had no significant difference with SX (*P* = 0.907). The SX and PC were also significantly different (*P* < 0.001).

As shown in Tables 2 and 3, in GCJ group after all stain removal methods, *ΔE* reduced to a clinically acceptable level but in other groups, the baseline color was not obtained.

## 4. Discussion

The color change of the dental restorative materials is often measured by Easyshade spectrophotometer according to the CIE *L*^*∗*^*a*^*∗*^*b*^*∗*^ color space, which was also used in the present study. Evidence shows that the acceptable *ΔE* is 0, which means a complete reversal of discolored composite to its baseline original color [13]. However, this can only be achieved in an ideal condition. According to Al-Nahedh and Awliya [12], the acceptable color change threshold is *ΔE* = 3.3.

Composite resin discoloration is caused by intrinsic and extrinsic factors. The intrinsic discoloration is the alteration of the resin matrix and the interface of the matrix and fillers that is due to a lack of enough polymerization or immersion in water for a long time. Extrinsic discoloration is staining by adsorption or absorption of colorants from exogenous sources [14]. To reduce extrinsic discoloration, the superficial roughness of the composite resin should be minimized, which is related to the size of its filler particles [15]. Note that resin composite absorbs water; it can also absorb other liquids, including beverages that cause color change in the deep portions of the restoration, so materials that have more water sorption will experience more discoloration in beverages. The water sorption occurs in the organic phase and the interface of the matrix and fillers and not in the filler particles [16, 17]. The amount of water that composite resins can absorb depends on the hydrophilicity of the matrix, filler composition, and the bonding quality between matrix and fillers [18].

In the present study, Filtek Z350 had more discoloration than Gradia Direct. A similar result has been reported in previous studies [19, 20] that showed nanohybrid composite resins had less color stability than microhybrid composite resins.

It has been shown that during polishing, filler particles are plucked out and make voids; therefore, in nanohybrid composite resins, smaller particles produce smaller voids which makes them experience less color change compared to microhybrid composite resins [21]. But the greater surface-to-volume ratio of nanoparticles provides a larger area of hydrophilic silane and increases water sorption. Furthermore, nanohybrid composite resins contain nanoclusters that are partially calcined irregular porous structures and have a high surface-to-volume ratio that causes various interfacial properties at the resin-filer interface [22]. In the study of Nasim et al. [23], Filtek Z350 had the most color change. They stated that it could be due to the composition of the resin matrix and the porosity of the nanoclusters and glass fillers. Cinelli et al. [24] concluded that the particles of the nanoclusters are not individually silanized and resulted in more water and pigment infiltration in nanohybrid composite resins.

Due to the presence of hydrophilic monomers, the composition and structure of the matrix can affect color stability. For instance, UDMA-based monomers are more susceptible to discoloration due to low viscosity, lower water sorption, and better polymerization compared to other methacrylate-based monomers [25]. This fact can justify the less color change of Gradia because, as shown in Table 4, it contains UDMA.

There was no significant difference in the color change with two solutions in the composite samples in general and in the Gradia samples, but in the Filtek Z350 samples, the color change was more with sour cherry juice than with coffee. Topcu et al. [25] demonstrated that the most staining solution for Filtek Z250 was the red wine, but for Filtek Supreme was granule lemon juice. These results show the important role of the composition of the staining solution on the color changes. Therefore, the difference in the staining capacity of solutions in different composite resins is not the same. The quality, particle size, and solubility of the stain, the concentration of the staining solution, and the interaction of stain with restorative material can be the contributing factors affecting the amount of composite resin color change [19].

The resin polymers and colorants have different polarities. If the polarity of the resin matrix is compatible with the colorant, the absorption of colorant into the organic phase of the composite resin material increases. Modification of the resin polymer polarities may improve the color stability of composite resins [8].

Coffee contains tannin, caffeine, and chlorogenic acid, which are less polar and water-soluble polyphenols and penetrate deep into the composite resin material because these colorants are compatible with the polymer matrix [26].

Sour cherry juice contains sugars, organic acids, mainly malic and malonic acids, and polyphenols such as flavonols, chlorogenic acids, and anthocyanins [27, 28]. The last one includes colored water-soluble pigments that are responsible for the color of many fruits and vegetables [29].

The PH range of the coffee is from 4.9 to 5.2, and for the sour cherry juice, it is between 3.2 and 3.3. As the pH decreases, the sorption and solubility of the material increase, which leads to surface degradation and softening that causes more surface roughness of the material. The rougher surface has lower optical reflection and is prone to erosion and discoloration [30, 31].

Bleaching involves a chemical reaction of oxidation–reduction, oxidizing the pigments present on the tooth and reducing the bleaching material [32]. The hydrogen peroxide gel releases free radicals that are able to penetrate the tooth structure. In this way, oxidation–reduction reactions are triggered, which break down the chromophores into smaller, colorless, and easily removable compounds [33]. These peroxide-based systems are expected to be better than polishing methods that just wearing the surface because of their ability to penetrate the composite subsurface and break down the pigment molecules. This issue was not proven in the present study.

This study evaluated the effectiveness of four whitening methods in removing stains from Gradia Direct and Filtek Z350 following exposure to sour cherry juice and coffee. The results demonstrated that SX performed significantly better than PC and was superior or equal to bleaching methods in all groups. PC was the least effective method except for GCJ, which was equal to OB, and ZCO, which was equal to HB. In Gradia, HB was significantly better than OB in both groups, and in Z350, HB was similar to OB in ZCJ group and less effective than OB in ZCO group. Also, in GCJ group after all stain removal methods, *ΔE* reduced to a clinically acceptable level but in other groups, the baseline color was not obtained.

Al-Nahedh and Awliya [12] showed HB was equal or superior to PC and OB, but in compared to SX, different results were obtained with different composite resins. SX also was similar or more effective compared to OB. Reinhardt et al. [34] showed that there were no statistically significant differences between PC and HB in whitening Amelogen Plus immersed in coffee and red wine. Hasani et al. [35] showed both home and office bleaching provided significant color changes in Filtek Z250, Filtek Z550, and Filtek Ultimate immersed in coffee and chlorhexidine, which was clinically acceptable.

Although bleaching procedures are effective in removing stains from the surface of the restoration, hydrogen peroxide can decrease the microhardness and cause softening and surface alteration of the composite resins, which makes them more susceptible to erosion and discoloration [30].

The abrasive particles in pumice are formed by the condensation of foamed volcanic glass to thin glass flakes. At the same time, aluminum oxide is the abrasive material in Sof-Lex discs. In order to have optimal abrasive efficacy, the abrasive agents should be harder than the composite fillers. Otherwise, the abrasives only remove the resin matrix and leave the filler particles [36].

In this study, Sof-Lex discs were more effective than pumice paste to regain the baseline color of the composite resin. Polishing with pumice can effectively clean the discolored surfaces that have smaller and smoother filler particles [12].

## 5. Conclusions

1. Filtek Z350 had more discoloration than Gradia Direct.
2. There was no significant difference in the color change with two solutions in the composite samples in general and in the Gradia samples, but in the Filtek Z350 samples the color change was more with sour cherry juice than with coffee.
3. The different materials and solutions reacted differently to the four stain removal methods; Sof-Lex discs performed significantly better than pumice paste and was superior or equal to bleaching methods in all groups. In GCJ group after all stain removal methods, *ΔE* reduced to a clinically acceptable level.

## Figures and Tables

**Table 1:** Means ± SD of color change of composite resins after immersion in staining solutions compared with baseline (*ΔE*_1_).

Composite resin	Staining solution	
	Sour cherry juice	Coffee
Gradia	9.98 ± 2.36^Aa^	12.43 ± 2.74^Aa^
Filtek Z350	25.47 ± 7.49^Ab^	23.53 ± 3.08^Bb^

Means labeled with the same uppercase letters are not significantly different, as compared in rows. Means labeled with the same lowercase letters are not significantly different, as compared in columns.

**Table 2:** Means ± SD of color change after stain removal compared with baseline (*ΔE*_2_) in Gradia.

Staining solution	Stain removal procedure			
	HB	OB	SX	PC
Sour cherry juice (GCJ)	1.93 ± 0.74^Aa^	2.82 ± 0.36^Ba^	2.09 ± 0.34^Aa^	3.17 ± 0.65^Ba^
Coffee (GCO)	3.32 ± 0.43^Aa^	4.73 ± 0.78^Ba^	3.83 ± 0.63^ABa^	6.46 ± 1.34^Ca^

Means labeled with the same uppercase letters are not significantly different, as compared in rows. Means labeled with the same lowercase letters are not significantly different, as compared in columns.

**Table 3:** Means ± SD of color change after stain removal compared with baseline (*ΔE*_2_) in Filtek Z350.

Staining solution	Stain removal procedure			
	HB	OB	SX	PC
Sour cherry juice (ZCJ)	7.60 ± 1.81^Aa^	4.72 ± 1.64^Aa^	4.11 ± 3.09^Aa^	13.59 ± 3.77^Ba^
Coffee (ZCO)	9.65 ± 1.87^Aa^	5.59 ± 0.90^Ba^	4.93 ± 1.23^Ba^	11.23 ± 3.40^Aa^

Means labeled with the same uppercase letters are not significantly different, as compared in rows. Means labeled with the same lowercase letters are not significantly different, as compared in columns.

**Table 4:** Materials used.

Product	Composition/type	Manufacturer/producer
Composite resin		

Filtek Z350	Nanohybrid Resin matrix: Bis-PMA, DUDMA, Bis-GMA, TEGDMA Filler: 82 w%/60 v%, including 0.6–1.4 *µ*m ZrO_2_/SiO_2_ nanoclusters, SiO_2_ nanofillers Shade: XWB	3M ESPE, St. Paul, MN, USA

Gradia Direct Anterior	Microhybrid Resin matrix: UDMA, dimethacrylate comonomers Filler: 73 w%/64 v%, including silica, 0.85 *µ*m prepolymerized fillers Shade: XBW	GC, Corp, Tokyo, Japan

Stain removal procedure		

Home bleaching (HB)	HOME WHITENING 16% carbamide peroxide	Whitesmile GmbH, Germany

Office bleaching (OB)	POWER WHITENING YF 40% hydrogen peroxide	Whitesmile GmbH, Germany

Sof-Lex discs (SX)	Aluminum oxide-coated abrasive disc medium (40 *µ*m)—fine (24 *µ*m)—*x*-fine (8 *µ*m)	3M ESPE, St. Paul, MN, USA

Pumice (PC)	Pumice powder medium	Cina, Iran

## Data Availability

The data used to support the findings of this study are available on request.

## Abbreviations

CIE: Commission International De l'Eclairage
XBW: Extra bleach white
XWB: Extra white body
GCJ: Gradia Direct specimens immersed in sour cherry juice
GCO: Gradia Direct specimens immersed in coffee
ZCJ: Filtek Z350 specimens immersed in sour cherry juice
ZCO: Filtek Z350 specimens immersed in coffee
SX: Polishing with Sof-Lex discs
PC: Polishing with pumice paste
HB: Home bleaching
OB: Office bleaching
Bis-GMA: Bisphenol A-glycidyl methacrylate
UDMA: Urethane dimethacrylate
TEGDEMA: Triethylene glycol dimethacrylate
Bis-PMA: 2,2-Bis-(4-(3-methacryloxypropoxy)phenyl)propane
DUDMA: Diurethane dimethacrylate.

## References

[1] Falkensammer F., Arnetzl G. V., Wildburger A., Freudenthaler J. (2013). Color stability of different composite resin materials. *The Journal of Prosthetic Dentistry*.

[2] Samorodnitzky-Naveh G. R., Grossman Y., Bachner Y. G., Levin L. (2010). Patients’ self-perception of tooth shade in relation to professionally objective evaluation. *Quintessence International*.

[3] Mutlu-Sagesen L., Ergün G., Özkan Y., Semiz M. (2005). Color stability of a dental composite after immersion in various media. *Dental Materials Journal*.

[4] Heimer S., Schmidlin P. R., Stawarczyk B. (2017). Discoloration of PMMA, composite, and PEEK. *Clinical Oral Investigations*.

[5] Villalta P., Lu H., Okte Z., Garcia-Godoy F., Powers J. M. (2006). Effects of staining and bleaching on color change of dental composite resins. *The Journal of Prosthetic Dentistry*.

[6] Bagheri R., Burrow M. F., Tyas M. (2005). Influence of food-simulating solutions and surface finish on susceptibility to staining of aesthetic restorative materials. *Journal of Dentistry*.

[7] (2004). Direct composite restorations: extended use in anterior and posterior situations. *Clinical Oral Investigations*.

[8] Ren Y.-F., Feng L., Serban D., Malmstrom H. S. (2012). Effects of common beverage colorants on color stability of dental composite resins: the utility of a thermocycling stain challenge model in vitro. *Journal of Dentistry*.

[9] Mundim F. M., Garcia L. D. F. R., Pires-de-Souza F. D. C. P. (2010). Effect of staining solutions and repolishing on color stability of direct composites. *Journal of Applied Oral Science*.

[10] Türkün L. S., Türkün M. (2004). Effect of bleaching and repolishing procedures on coffee and tea stain removal from three anterior composite veneering materials. *Journal of Esthetic and Restorative Dentistry*.

[11] Kihn P. W. (2007). Vital tooth whitening. *Dental Clinics of North America*.

[12] Al-Nahedh H. N., Awliya W. Y. (2013). The effectiveness of four methods for stain removal from direct resin-based composite restorative materials. *The Saudi Dental Journal*.

[13] Seghi R. R., Hewlett E. R., Kim J. (1989). Visual and instrumental colorimetric assessments of small color differences on translucent dental porcelain. *Journal of Dental Research*.

[14] Awliya W. Y., Al-Alwani D. J., Gashmer E. S., Al-Mandil H. B. (2010). The effect of commonly used types of coffee on surface microhardness and color stability of resin-based composite restorations. *The Saudi Dental Journal*.

[15] Kolbeck C., Rosentritt M., Lang R., Handel G. (2006). Discoloration of facing and restorative composites by UV-irradiation and staining food. *Dental Materials*.

[16] Szczesio-Wlodarczyk A., Sokolowski J., Kleczewska J., Bociong K. (2020). Ageing of dental composites based on methacrylate resins—a critical review of the causes and method of assessment. *Polymers*.

[17] Garcia P. P. N. S., Neto E. R., dos Santos P. A., Campos J. Á. D. B., Dibb R. G. P. (2008). Influence of surface sealant on the translucency of composite resin: effect of immersion time and immersion media. *Materials Research*.

[18] Berger S. B., Palialol A. R. M., Cavalli V., Giannini M. (2009). Characterization of water sorption, solubility and filler particles of light-cured composite resins. *Brazilian Dental Journal*.

[19] Malhotra N., Shenoy R. P., Acharya S., Shenoy R., Mayya S. (2011). Effect of three indigenous food stains on resin-based, microhybrid-, and nanocomposites. *Journal of Esthetic and Restorative Dentistry*.

[20] Yazici A. R., Çelik Ç., Dayangaç B., Ozgünaltay G. (2007). The effect of curing units and staining solutions on the color stability of resin composites. *Operative Dentistry*.

[21] Ferooz M., Bagheri R., Jafarpour D., Burrow M. F. (2020). Physical properties of nanohybrid and microhybrid resin composites subjected to an acidic environment: a laboratory study. *Operative Dentistry*.

[22] Curtis A. R., Shortall A. C., Marquis P. M., Palin W. M. (2008). Water uptake and strength characteristics of a nanofilled resin-based composite. *Journal of Dentistry*.

[23] Nasim I., Neelakantan P., Sujeer R., Subbarao C. V. (2010). Color stability of microfilled, microhybrid and nanocomposite resins—an in vitro study. *Journal of Dentistry*.

[24] Cinelli F., Russo D. S., Nieri M., Giachetti L. (2022). Stain susceptibility of composite resins: pigment penetration analysis. *Materials*.

[25] Topcu F. T., Sahinkesen G., Yamanel K., Erdemir U., Oktay E. A., Ersahan S. (2009). Influence of different drinks on the colour stability of dental resin composites. *European Journal of Dentistry*.

[26] Telang A., Narayana I. H., Madhu K. S., Kalasaiah D., Ramesh P., Nagaraja S. (2018). Effect of staining and bleaching on color stability and surface roughness of three resin composites: an *in vitro* study. *Contemporary Clinical Dentistry*.

[27] Sokół-Łętowska A., Kucharska A. Z., Hodun G., Gołba M. (2020). Chemical composition of 21 cultivars of sour cherry (*Prunus cerasus*) fruit cultivated in Poland. *Molecules*.

[28] Mayta-Apaza A. C., Daya M., Franck C. (2019). Tart cherries and health: current knowledge and need for a better understanding of the fate of phytochemicals in the human gastrointestinal tract. *Critical Reviews in Food Science and Nutrition*.

[29] Khoo H. E., Azlan A., Tang S. T., Lim S. M. (2017). Anthocyanidins and anthocyanins: colored pigments as food, pharmaceutical ingredients, and the potential health benefits. *Food & Nutrition Research*.

[30] Al Ahmari N. M., Alahmari M. A., Al Moaleem M. M. (2022). Physical, optical, and mechanical properties of ceramic materials after coffee immersion and evaluation of cleaning impact with different oral hygiene tools. *International Journal of Environmental Research and Public Health*.

[31] Fiorillo L., Cervino G., Herford A. S., Laino L., Cicciù M. (2020). Stannous fluoride effects on enamel: a systematic review. *Biomimetics*.

[32] Saati K., Sheikhi S., Esnaashari E., Valizadeh S. (2019). The effects of three bleaching agents on tooth discoloration caused by mineral trioxide aggregate. *Iranian Endodontic Journal*.

[33] Fiorillo L., Laino L., De Stefano R. (2019). Dental whitening gels: strengths and weaknesses of an increasingly used method. *Gels*.

[34] Reinhardt J. W., Balbierz M. M., Schultz C. M., Simetich B., Beatty M. W. (2019). Effect of tooth-whitening procedures on stained composite resins. *Operative Dentistry*.

[35] Hasani E., Baghban A. A., Sheikh-Al-Eslamian S. M., Sadr A. (2019). Effect of bleaching on color change of composite after immersion in chlorhexidine and coffee. *Journal of Conservative Dentistry*.

[36] Barakah H. M., Taher N. M. (2014). Effect of polishing systems on stain susceptibility and surface roughness of nanocomposite resin material. *The Journal of Prosthetic Dentistry*.

